# Mechanistic insights into the potential role of botanical drugs and plant-derived metabolites in mitigating oxidative stress in varicocele

**DOI:** 10.3389/fphar.2026.1720260

**Published:** 2026-09-28

**Authors:** Anmin Wang, Elena Colonnello, Dongyue Ma, Hongyuan Chang, Hao Wang, Hui Lv, Di Sun, Haoyu Zhang, Wenxiao Yu, Fu Wang

**Affiliations:** 1 Department of Andrology, Xiyuan Hospital of China Academy of Chinese Medical Sciences, Beijing, China; 2 Endocrinology and Medical Sexology (ENDOSEX), Department of Systems Medicine, University of Rome Tor Vergata, Rome, Italy; 3 Department of Andrology, Xiyuan Hospital of Clinical Medical College of Beijing University of Chinese Medicine, Beijing, China; 4 Department of Rheumatology, Xiyuan Hospital of Clinical Medical College of Beijing University of Chinese Medicine, Beijing, China

**Keywords:** botanical drug, oxidative stress, plant-derived metabolites, therapeutic mechanisms, varicocele

## Abstract

Varicocele (VC) is a prevalent male urogenital disorder associated with scrotal discomfort, impaired semen quality, and endocrine dysregulation, in which oxidative stress (OS) is repeatedly implicated as a key pathogenic axis linking venous stasis, hypoxia, hyperthermia, inflammation, mitochondrial dysfunction, and germ-cell injury. This review critically evaluates experimental and clinical evidence on defined botanical drugs and plant-derived metabolites investigated in VC or VC-relevant OS models, with an emphasis on mechanistic plausibility and translational limitations. Across *in vivo* VC models, toxin-/heat-/hypoxia-induced spermatogenic injury models, and selected clinical studies, multiple interventions have been reported to improve semen parameters, testicular histology, and redox indices (e.g., ROS, MDA, SOD/CAT/GPx, GSH/GSSG), alongside modulation of apoptosis and stress responses. Reported molecular nodes converge on redox- and stress-responsive pathways, including Keap1–Nrf2–ARE/HO-1, PI3K–Akt, MAPK/JNK–p38, HIF-1–related signaling, ER-stress responses, mitochondrial apoptosis (e.g., Bax/Bcl-2/caspases), and, in some models, ferroptosis-related regulation. However, the overall evidence base is constrained by heterogeneity in models and endpoints, limited extract/compound chemical characterization and pharmacological standardization, frequent reliance on *in silico* predictions, small or non-randomized clinical cohorts, and incomplete reporting of taxonomic and product quality details. Future work should prioritize rigorously designed, adequately powered clinical trials with standardized outcomes, well-defined interventions, and mechanism-aligned biomarkers to clarify which botanical drugs/metabolites offer clinically meaningful benefit as adjuncts in VC management.

## 1 Introduction

Varicocele (VC) is a common male urogenital condition characterized by abnormal dilation of the pampiniform plexus. Clinically, it is most often associated with three outcomes: infertility, scrotal pain/discomfort, and testosterone dysregulation, with the link to male infertility receiving the greatest attention (Minhas et al., 2021). Accumulating experimental and clinical evidence over the past few decades has identified oxidative stress (OS) as a central pathological process linking venous stasis, scrotal hyperthermia, hypoxia, inflammation, and impaired spermatogenesis in VC (Jensen et al., 2017). Excessive reactive oxygen species (ROS) generation and insufficient antioxidant defense contribute to lipid peroxidation, DNA damage, mitochondrial dysfunction, and germ cell apoptosis. Accordingly, redox-sensitive signaling pathways, including nuclear factor erythroid 2-related factor 2 (Nrf2)-mediated antioxidant responses, phosphatidylinositol 3-kinase (PI3K)/protein Kinase B (Akt), mitogen-activated protein kinase (MAPK), and inflammatory cascades, have emerged as mechanistically relevant targets in VC.

The benefit of surgical intervention in cases of subclinical VC or in patients without fertility concerns remains controversial, particularly regarding ROS levels, which can be variable (Persad et al., 2021; Baazeem et al., 2011; El-Hamd et al., 2016). Recently, interest has grown in the use of non-surgical therapies. Botanical drugs have been used used in traditional Chinese medicine (TCM) as potential sources of bioactive secondary metabolites capable of modulating OS-related pathways for VC (Marini et al., 2022; Garolla et al., 2022; Cao et al., 2025). Agents such as aescin and diosmetin have demonstrated effects on capillary permeability, lymphatic drainage, and venous tone, which may contribute to reduced edema, pain, and improved microcirculation (Ye and Shen, 2017; Dogan et al., 2014). However, much of the existing literature remains descriptive, frequently extrapolating generalized “antioxidant” or “protective” effects without sufficient pharmacological rigor. In this review, we critically assess the experimental and clinical evidence regarding defined botanical drugs and plant-derived metabolites investigated in the context of VC or VC-relevant OS models, with the aim of identifying both mechanistic insights and key limitations that must be addressed in future research.

## 2 Oxidative stress: molecular mechanisms and implications for VC

OS is an imbalance between ROS production and antioxidant defenses, and is repeatedly implicated as a central mediator of testicular dysfunction in VC (Morielli and O'Flaherty, 2015; Agarwal et al., 2009; Agarwal et al., 2012; Cho et al., 2016). Venous stasis, hypoxia, inflammation, and localized hyperthermia in VC favor sustained ROS generation and compromise endogenous redox control, promoting DNA strand breaks, protein dysfunction, lipid peroxidation, and disrupted cellular homeostasis, resulting in impaired spermatogenesis and clinically significant VC manifestations (Wang et al., 2022; Talebi-Yazdabadi et al., 2021). Mechanistically, OS in VC should be viewed as dysregulation of redox-sensitive signaling networks and mitochondria-driven cell death programs rather than solely as altered enzyme activities (Zhang et al., 2020) (See Figure 1).

**FIGURE 1:**
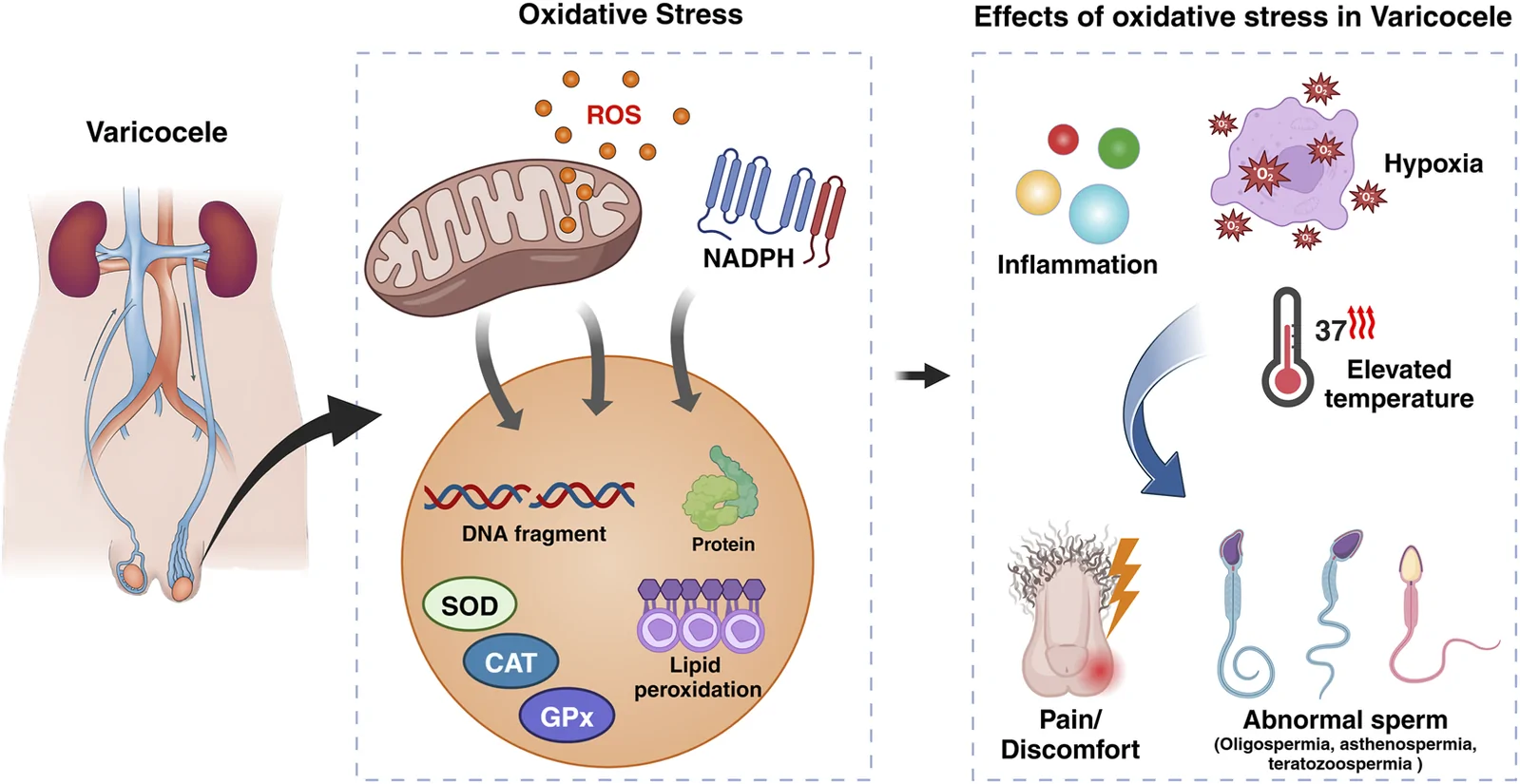
Effects of Oxidative Stress in Varicocele. Varicocele-associated venous stasis, tissue hypoxia and scrotal hyperthermia promote increased reactive oxygen species production—principally from mitochondrial respiratory chain dysfunction and NADPH—which induces DNA fragmentation, protein oxidation and lipid peroxidation in testicular cells and overwhelms endogenous antioxidant defenses (SOD, CAT, GPx); concomitant local inflammation amplifies this oxidative injury, ultimately producing scrotal pain/discomfort and adverse semen parameters including oligospermia, asthenozoospermia and teratozoospermia.

### 2.1 Elevated testicular temperature

Physiological thermoregulation maintains testicular temperature 2 °C–4 °C below core body temperature in healthy males, a critical requirement for normal sperm production, given the testes’ marked thermosensitivity (Mariotti et al., 2011). In VC, venous stasis and metabolite accumulation promote excessive generation of reactive oxygen and nitrogen species, reflected by elevated levels of malondialdehyde (MDA) and 4-hydroxynonenal-modified proteins, along with dysregulated expression of heat- and redox-responsive genes, including heme oxygenase 1 (HMOX1) and glutathione peroxidase 1 (GPX1), which in turn contribute to localized heat retention and elevated testicular temperature (Fang et al., 2023; Shiraishi et al., 2010; Paul et al., 2009). Importantly, heat-induced redox imbalance converges on mitochondrial dysfunction and activation of pro-apoptotic signaling pathways, providing a mechanistic rationale for therapeutic strategies aimed at preserving mitochondrial integrity or enhancing adaptive antioxidant defenses (Hassun et al., 2005).

### 2.2 Inflammatory reaction

ROS and inflammation form a feed-forward loop in VC: ROS promote cytokine production and inflammasome activation, while recruited leukocytes generate additional ROS (Wang et al., 2025; Minas et al., 2023; Yang et al., 2025). Within this environment, immune cells function as critical immune regulation relays, secreting elevated concentrations of pro-inflammatory cytokines, including interleukin-1 (IL-1), IL-6, IL-8, and tumor necrosis factor-α (TNF-α) levels (Leuti et al., 2020; Razi et al., 2012). Among these mediators, IL-6 enhances ROS generation in the testicular environment while concurrently reducing overall antioxidant capacity. In parallel, ROS serves as a critical mediator in activating NLRP3 inflammasome, leading to caspase-1 activation and subsequent impairment of spermatogenesis and testosterone synthesis (Poli et al., 2022; Pisoschi and Pop, 2015). Crosstalk between oxidative and inflammatory pathways further induces inducible nitric oxide synthase (iNOS), reinforcing a vicious cycle of oxidative and nitrosative stress. Clinical investigations further validate this, as post-varicocelectomy analyses demonstrate the reduction of caspase-1 and IL-1β, indicating attenuation of inflammasome-driven inflammation following correction of venous stasis (Ata-Abadi et al., 2020). Sustained inflammatory stress also compromises blood–testis barrier integrity, promoting antisperm antibody formation and reduced sperm viability, thereby linking OS-driven inflammation to VC impairment (Gow, 2005; Panner Selvam et al., 2019).

### 2.3 Hypoxia

Varicocele-induced venous stasis produces a hypoxic testicular microenvironment characterized by diminished partial pressure of oxygen, ROS overproduction, and redox imbalance (Aitken and Curry, 2011). This chronic hypoxia regulates hypoxia-inducible factor 1 alpha (HIF-1α), a subunit predominantly expressed in spermatogenic cell cytoplasm and vascular endothelium. While transient HIF-1α stabilization offers short-term cytoprotection, sustained accumulation under pathological hypoxia triggers p53-mediated apoptosis and disrupts mitochondrial integrity via impaired HSP synthesis and metabolic reprogramming (Hu et al., 2015; Zhao et al., 2019; Greijer and van der Wall, 2004; Wang et al., 2010; Liang et al., 2015). Concurrently, this hypoxic/oxidative milieu perturbs the hypothalamic–pituitary–gonadal axis: VC patients frequently exhibit reduced inhibin B and increased gonadotropins (follicle-stimulating hormone/luteinizing hormone), reflecting impaired spermatogenesis and endocrine dyscrasia (Damsgaard et al., 2016; Li et al., 2021). These observations delineate an interdependent triad of hypoxia, OS, and endocrine dysfunction that underpins testicular damage in VC.

## 3 Chinese medicinal botanical drugs for varicocele: pharmacological basis and evidence assessment

In the paragraphs below we summarize commonly used botanical drugs investigated for VC-related OS. For each botanical drug we give (A) the full taxonomic name (with authority) and family (B) principal plant-derived metabolite(s) or active fractions and putative molecular targets, and (C) a concise critical appraisal of the experimental/clinical evidence. Authors and readers should consult Table 2 for detailed study-level information (See Tables 1, 2; Figure 2).

**TABLE 1:** Category, main plant metabolites, and chemical structures of botanical drugs relevant to varicocele.

Category	Botanical drugs	Pictures	Plant metabolites	Structure
Tonifying Qi	*Astragalus mongholicus* Bunge (Huangqi)	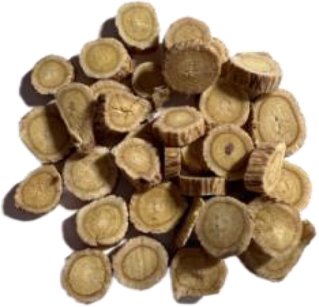	*Astragalus mongholicus* polysaccharide	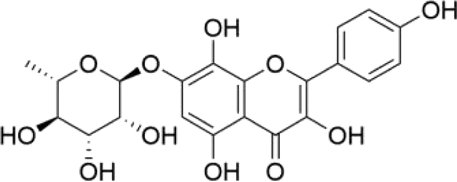
			Astragaloside IV	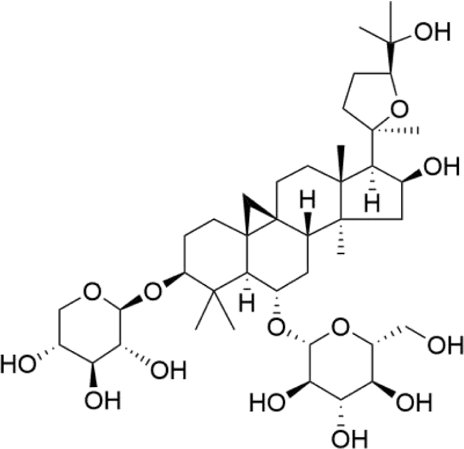
	*Panax ginseng* C.A.Mey. (Renshen)	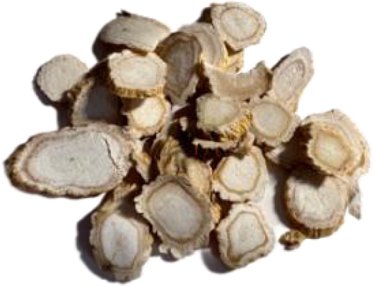	Ginsenoside Rb1	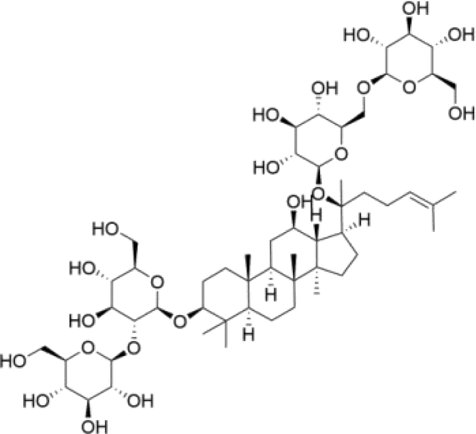
			Ginsenoside Rg3	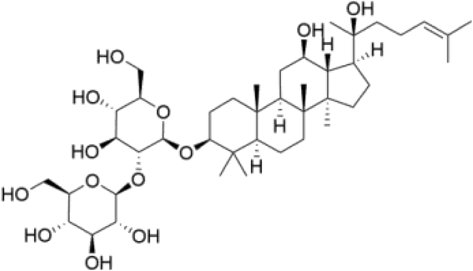
	*Rhodiola rosea* L. (Hongjingtian)	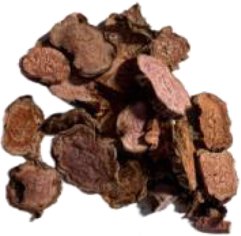	Rhodionin	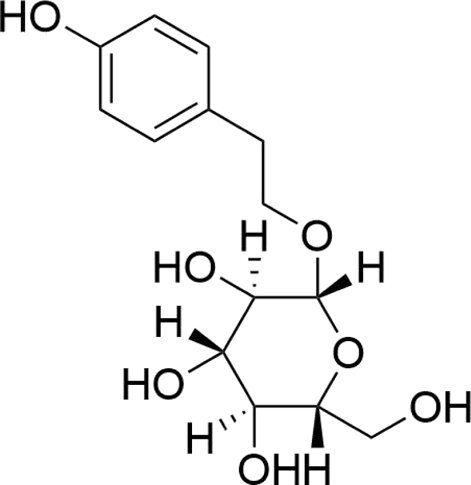
			Salidroside	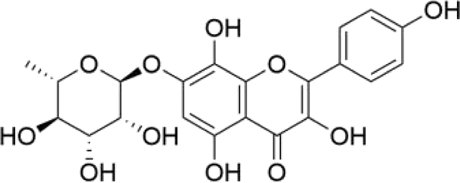
Reinforcing yang	*Cuscuta chinensis* Lam (Tusizi)	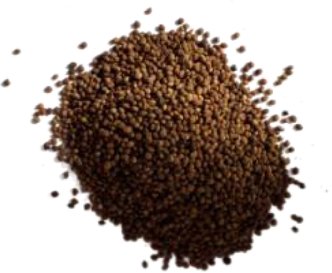	Quercetin	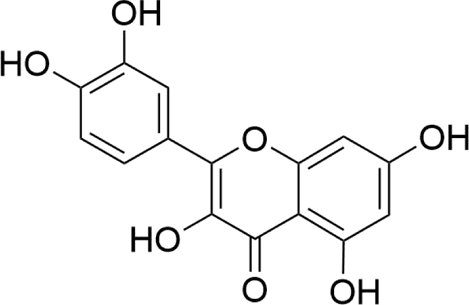
			Kaempferia galanga Linn	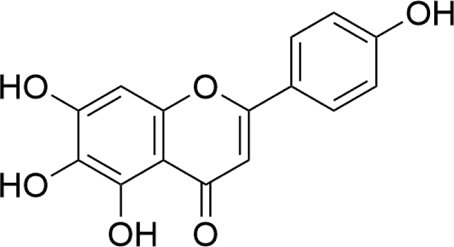
			Isorhamnetin	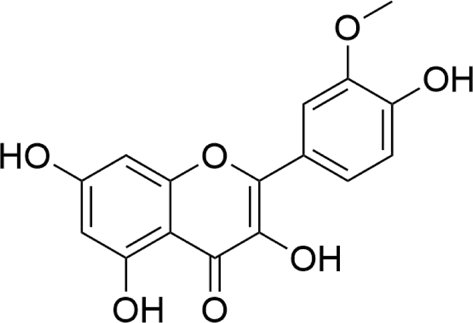
			Mannose	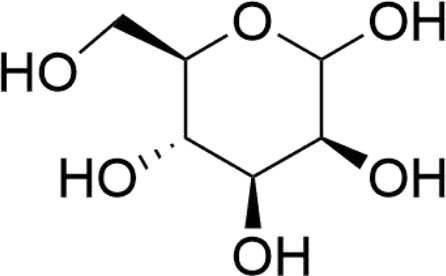
	*Gynochthodes officinalis* (F.C.How) Razafim. & B.Bremer (Bajitian)	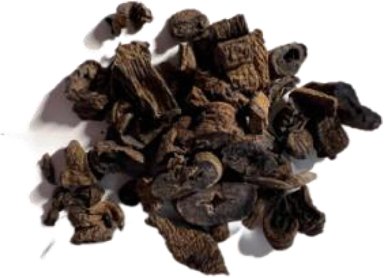	L-Arabinose	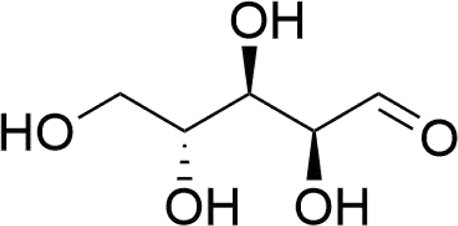
			Galactose	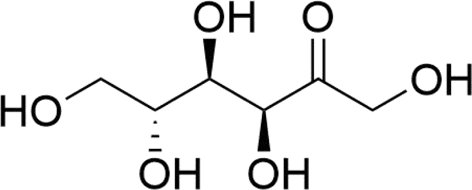
	*Epimedium brevicornu* Maxim. (Yinyanghuo)	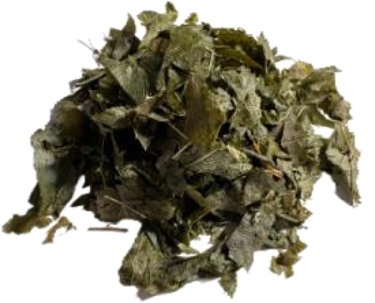	Mannose	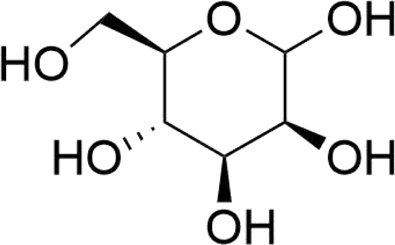
			Rhamnose	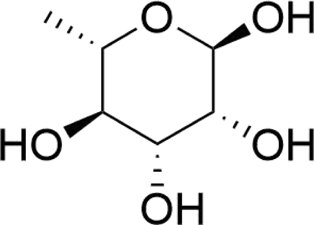
			Galacturonic acid	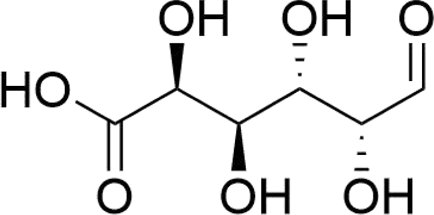
			Icartin	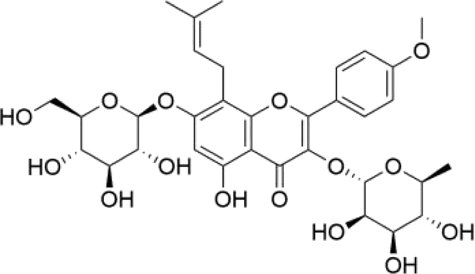
			Icariin	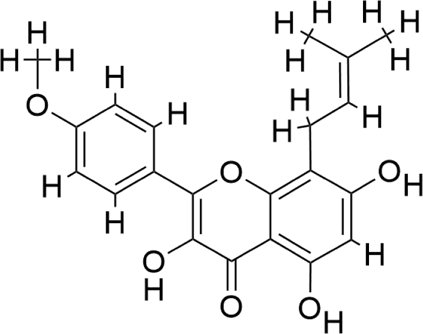
Tonifying yin	*Schisandra chinensis* (Turcz.) Baill. (Wuweizi)	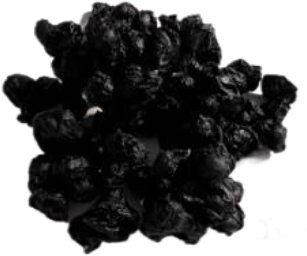	Schisandrin B	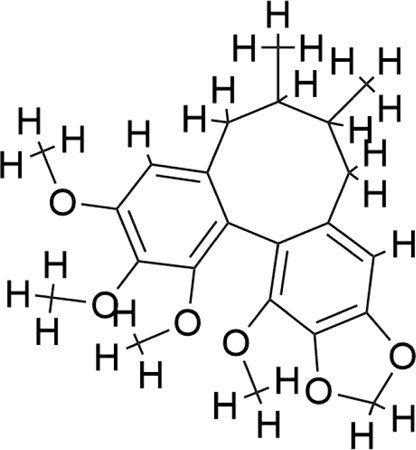
Activating blood	*Lycium chinense* Mill (Gouqi)	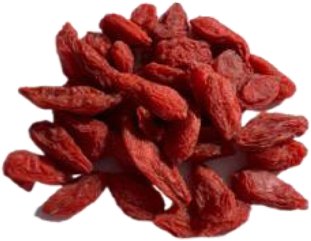	Galactose	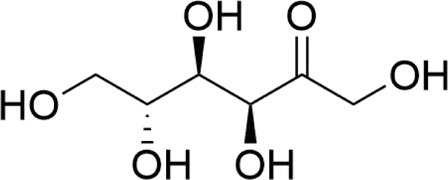
			L-Arabinose	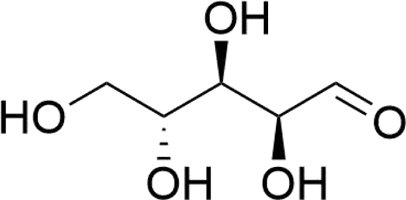
			Rhamnose	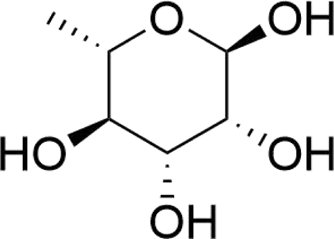
Activating blood	*Curcuma longa* L. (Jianghuang)	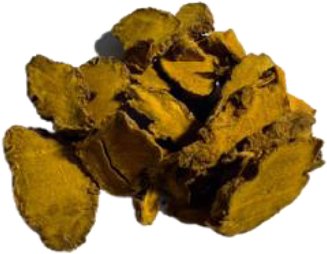	Curcumin	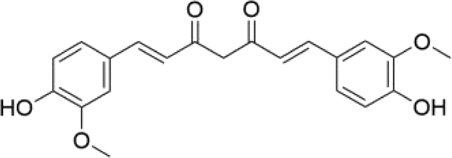

**TABLE 2:** Targets/Pathways and reported effects of botanical drug metabolites relevant to varicocele.

Botanical drug	Metabolites	Ref.	Type of study	Model/Subject	Key outcome measure	Targets/Pathways	Effects
*Astragalus mongholicus* Bunge (Huangqi)	*Astragalus mongholicus* polysaccharide	Lv et al. (2021)	In silico (network pharmacology; molecular docking)	TCMSP and disease-target databases; PPI/GO/KEGG; docking	Predicted active metabolites, targets and enriched pathways; docking affinity	PI3K-Akt (VEGFA, AKT1, IL6, CASP9, TP53, EGFR, PTEN, BCL2); HIF-1 (NOS2, HMOX1, HIF1A, VEGFA, IL6, AKT1)	Oxidative stress
	Astragaloside IV	Cai et al. (2013)	*In vivo*	Rodent VC model	Blood exposure; testicular microstructure	HO-1-related antioxidant response	Oxidative stress Apoptosis
		Li et al. (2023)	*In vitro*	TM3 mouse Leydig cells	Cell viability; ROS; apoptosis rate; Caspase-9/Bax expression	Intrinsic apoptosis (Caspase-9, Bax)	Oxidative stress Apoptosis
*Panax ginseng* C.A.Mey. (Renshen)	Ginsenoside Rb1	Wang et al. (2018)	*In vivo*	D-galactose-induced aging mouse model	Spermatogenic parameters; antioxidant indices; apoptosis markers	Keap1-Nrf2 signaling; apoptosis regulation (Bcl-2, Caspase-3)	Oxidative stress
		Huang et al. (2024)	*In vivo* + *in vitro*	C57BL/6J mice heat-stress model GC-2spd (ts) heat-stress model	Testis index; histology; sperm motility; OS markers; apoptosis; Nrf2-axis readouts	Keap1-Nrf2-ARE (HO-1, NQO1, GCLC, GCLM); Bax/Bcl-2/Caspase-3 axis	Oxidative stress Apoptosis
	Ginsenoside Rg3	Ma (2024)	*In vivo* + *in vitro*	ICR mice DEHP-induced spermatogenic impairment; TM3 Leydig cells + TM4 Sertoli cells MEHP-induced injury	Sperm count; testis histology; serum LH/testosterone; antioxidant status; testosterone-synthesis proteins	Sperm count; testis histology; serum LH/testosterone; antioxidant status; testosterone-synthesis proteins	Oxidative stress Ferroptosis
*Rhodiola rosea* L. (Hongjingtian)	Salidroside	Song (2023)	*In vitro*	GC-1 mouse spermatogonial cells hypoxia oxidative-injury model	Cell viability; OS markers; mitochondrial function; apoptosis rate; apoptosis proteins	Mitochondrial apoptosis (cyt c-Bax/Bcl-2-caspase-3)	Oxidative stress Apoptosis
	Rhodiola Polysaccaride	Yang et al. (2016)	*In vitro*	Frozen–thawed boar sperm	Sperm motility; antioxidant enzymes; lipid peroxidation marker	Antioxidant enzymes (SOD, GSH-Px); lipid peroxidation (MDA)	Oxidative stress
*Cuscuta chinensis* Lam (Tusizi)	—	Soni et al. (2018)	*In vivo*	Rat left renal vein partial ligation varicocele model	Left testis weight; serum hormones; sperm motility/count; ER-response proteins; oxidative stress markers	ROS-related ER stress/UPR (ER-response proteins)	Oxidative stress
	*Cuscuta Chinensis* flavonoids	Liu et al. (2018)	*In vitro*	B16F10 melanoma cells	Antioxidant capacity; melanogenesis readouts; cytotoxicity; molecular weight distribution	Melanogenesis regulators (MITF, tyrosinase, TRP-1)	Oxidative stress
		Feng et al. (2025)	*In vivo*	*Tripterygium wilfordii* glycosides–induced oligoasthenospermia	Testicular index; sperm concentration; total motility; testis histology	Keap1/Nrf2/HO-1 pathway	Oxidative stress
​	​	Li (2022)	*In vivo* + *in vitro*	Balb/c mice cyclophosphamide testicular-injury model; TM3 acrolein-injury model	Body/testis weight; sperm count/viability; oxidative stress; serum testosterone; apoptosis proteins	ERK-Nrf2-ARE (p-ERK/ERK, Nrf2, NQO1, HO-1); apoptosis (Bcl-2, Bax, caspase-3)	Oxidative stress Apoptosis
*Gynochthodes officinalis* (F.C.How) Razafim. & B.Bremer (Bajitian)	—	Chang et al. (2008)	*In vitro*	TM3 (mouse Leydig) H_2_O_2_-induced oxidative stress model	Cell viability; DPPH scavenging; lipid peroxidation; testosterone; antioxidant enzymes	Antioxidant defense (SOD, CAT); lipid peroxidation	Oxidative stress
	*Gynochthodes officinalis* polysaccharide	Yu et al. (2024)	*In vivo*	Rat left renal vein partial ligation varicocele model	Testis/epididymis coefficients; seminiferous tubule diameter; sperm count; ROS; apoptosis; hormones	PTEN/PI3K/AKT pathway	Oxidative stress
		Zhang (2017)	*In vivo* + *in vitro*	Rat left renal vein partial ligation varicocele model TM4 (Sertoli) miR-181d-5p mimic overexpression	Testis/epididymis morphology; sperm count; differential miRNA (miR-181d-5p)	miR-181d-5p→PTEN; FAK-PI3K/Akt signaling	Oxidative stress
*Epimedium brevicornu* Maxim. (Yinyanghuo)	Icaritin	Huang et al. (2022)	*In vivo*	Rat left renal vein partial ligation varicocele model	Testis/epididymis indices; sperm concentration/motility; apoptosis; SOD/LDH-X/MDA	Nrf2/Keap1/HO-1 pathway	Oxidative stress Apoptosis
	Icariin	Zha et al. (2014a)	*In vivo*	Rat left renal vein partial ligation varicocele model	Epididymis histology; epididymal SOD/GSH-Px activities; MDA	Antioxidant defense (SOD, GSH-Px); lipid peroxidation (MDA)	Oxidative stress
		Jiang et al. (2024)	*In vivo*	KM mice adenine-induced oligoasthenospermia model	Sperm quality: Density, motility; serum hormones; iron deposition; oxidative stress	P53–SLC7A11–GPX4	Oxidative stress Ferroptosis
*Schisandra chinensis* (Turcz.) Baill. (Wuweizi)	Schisandrin B	Zhang et al. (2023)	*Ex vivo* + *in vitro*	Human sperm H_2_O_2_ oxidative-stress model	Progressive motility; sperm ROS; mRNA expression; IVF outcomes	SIRT1/Nrf2 pathway (SIRT1, Nrf2, SOD2, GSH-Px)	Oxidative stress
*Lycium chinense* Mill (Gouqi)	*Lycium chinense* polysaccharide	Xu (2021)	*In vitro*	GC-1spg (mouse spermatogonia) HX–XO oxidative-stress model	Cell viability; oxidative stress markers; apoptosis markers	Antioxidant defense (SOD, CAT, GSH); caspase-mediated apoptosis (Caspase-3, Caspase-9)	Oxidative stress Apoptosis
		Tian (2022)	*In vitro*	Porcine oocyte IVM + IVF embryo culture	IVF outcomes; polyspermy/monospermy; oocyte ROS	Nrf2 signaling; antioxidant genes (CAT, SOD1, GPX4); apoptosis genes (BCL2, BAX); peroxisome genes (PEX3/5/19, PHYH, PPARγ)	Oxidative stress
*Curcuma longa* L. (Jianghuang)	Curcumin	Liu (2024)	*In vivo* + *in vitro*	ICR mice heat-stress model GC-2 (mouse spermatocyte) heat-stress model	Testis/epididymis indices; sperm motility; apoptosis; oxidative stress; GC-2 oxidative/inflammatory markers	ATF3; apoptosis (Bax/Bcl-2; caspases); cytokines (TNF-α, IL-6, IL-4, IL-10)	Oxidative stress Inflammation
		Yang (2016)	*In vivo*	Kunming mice cyclophosphamide reproductive-toxicity model	Testis/epididymis indices; serum testosterone; sperm count; testis oxidative markers	Antioxidant defense (SOD, CAT, GSH, GSH-Px); lipid peroxidation (MDA)	Oxidative stress

**FIGURE 2:**
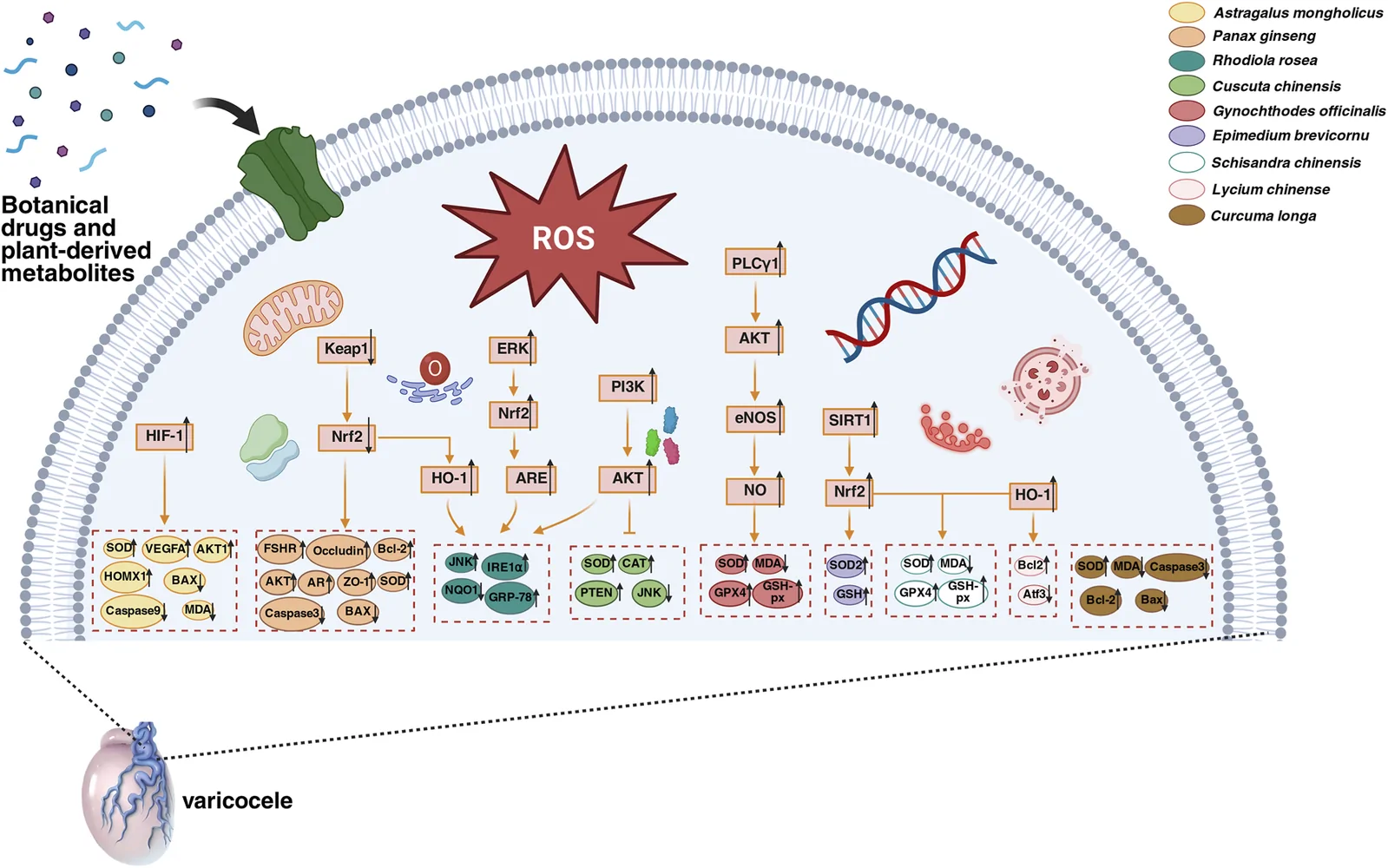
Botanical drug preparations used for varicocele management. Schematic overview of varicocele-associated oxidative stress signaling. Venous stasis/hypoxia promotes reactive oxygen species accumulation, mitochondrial dysfunction, and germ-cell injury through redox- and stress-responsive pathways (e.g., Nrf2, PI3K/AKT, MAPK, HIF-1). Representative botanical drugs/metabolites are mapped to their reported intervention nodes (antioxidant defense activation, mitochondrial protection, and attenuation of apoptosis/inflammation).

### 3.1 *Astragalus mongholicus* Bunge, Fabaceae (Huangqi)

*Astragalus mongholicus* Bunge is a medical plant whose dried root (Huangqi) has been used since it was first recorded in the Shennong’s Classic of Materia Medica (Shennong Bencao Jing), written in the Eastern Han dynasty (25–220 CE) (Su et al., 2021). In TCM, it is classically indicated to “tonify qi” (enhance vital energy) and “raise yang” (promote physiological activity), strengthen the body’s exterior to reduce sweating, and promote urination to relieve edema (Su et al., 2021). Huangqi and its derived metabolites are employed in treating various conditions, including cancer, cardiovascular diseases, hypertension, and diabetes mellitus (Liu et al., 2024a). Contemporary phytochemistry identifies over 400 metabolites, with dominant classes being water-soluble polysaccharides (20%–30% dry weight), flavonoids (15%–25%), and triterpenoid saponins (10%–18%), the latter including numerous astragalosides (e.g., astragalosides I–VIII, isoastragalosides), of which astragaloside IV is frequently used as a marker plant-derived metabolites (Fu et al., 2014; Tang and Huang, 2022; Chen et al., 2020).

Experimental studies have shown that huangqi-derived metabolites significantly reduce intracellular ROS, enhance superoxide dismutase (SOD) activity, and decrease MDA levels—key indicators of oxidative damage, indicating intrinsic antioxidant capacity; however, these effects have largely been characterized *in vitro* or in non-gonadal disease models, leaving their efficacy within the VC-altered testicular microenvironment insufficiently defined (Yao et al., 2024). Network pharmacology further suggests modulation of PI3K–Akt and HIF-1 signaling via targets such as Akt1, vascular endothelial growth factor A (VEGFA), and HIF1A, supporting a multi-target mechanism against hypoxia and apoptosis, although these interactions remain predominantly computational and require experimental confirmation in VC models (Lv et al., 2021). In experimental VC settings, Astragalus polysaccharides attenuated seminiferous tubular injury and reduced heme oxygenase 1 (HO-1)–derived carbon monoxide levels, a surrogate marker of hypoxia-associated germ cell damage, thereby partially restoring spermatogenic architecture (Cai et al., 2013). At the cellular level, astragaloside IV (10–100 mg L^−1^) suppressed H_2_O_2_-induced apoptosis in TM3 Leydig cells by stabilizing mitochondrial membrane potential and downregulating BCL-2-associated X protein (Bax) and caspase-9, providing mechanistic support for anti-apoptotic activity, albeit in a simplified OS model rather than VC-specific pathology (Li et al., 2023).

### 3.2 *Panax ginseng* C.A.Mey., Araliaceae (Renshen)

*Panax ginseng* C.A. Mey. (Araliaceae), also known as Asian or red ginseng, is a plant whose dried root and rhizome’s medical use (Renshen) was first recorded in the Shennong’s Classic of Materia Medica (Shennong Bencao Jing) (Yu et al., 2024). Renshen is classically employed in TCM to “tonify the original qi”, counter fatigue, promote fluid generation, and produce a calming effect (Chan, 2012). Phytochemical investigations identify ginsenosides, polysaccharides, amino acids, volatile oils and trace elements as principal metabolites; ginsenosides, particularly dammarane-type saponins (e.g., Rb1, Rb2, Rc, Rd, Rg3, Rh2), together with oleanolic- and ocotillol-type congeners are the most extensively studied bioactives, while ginseng polysaccharides account for roughly 5% of dry weight and contribute to the plant’s pharmacological profile (Liu Y. et al., 2021; Mancuso and Santangelo, 2017; Chen et al., 2020; Li and Zhang, 2016).

In VC, elevated levels of lipid peroxides in testicular tissues can damage spermatogenic cells and disrupt their subcellular membrane structures, leading to spermatogenic dysfunction. Ginsenosides exert antioxidant effects mainly by inhibiting lipid peroxidation and scavenging excess ROS. In a Wistar rat model of surgically induced VC, a panaxadiol-type ginsenoside fraction significantly reduced lipid peroxide levels in testicular tissue and serum, suggesting attenuation of lipid peroxidation–mediated injury. However, this evidence is limited to a single VC rodent model with an imbalanced group design, a chemically undefined ginsenoside mixture, and no pharmacokinetic characterization or comparison with established antioxidant controls (Xi et al., 1992; Xi et al., 1993). In a D-galactose–induced aging mouse model, ginsenoside Rg1 reduced testicular apoptosis and improved spermatogenic parameters, correlating with increased Bcl-2, decreased cleaved caspase-3, enhanced SOD activity, and reduced MDA levels (Wang et al., 2018). Likewise, in heat- and OS models relevant to VC-associated hyperthermia, ginsenosides (e.g., Rg1/Rb1) partially reversed Bax and caspase-3 upregulation, restored Bcl-2 expression, and modulated the kelch-like ECH-associated protein 1 (Keap1)-Nrf2 pathway. Nonetheless, these studies rely on simplified or non-VC models, with limited pharmacokinetics and pharmacodynamics (PK/PD) data, inconsistent extract standardization, and scarce evidence that active saponins reach effective concentrations within the hyperthermic testicular microenvironment (Hou et al., 2022; Huang et al., 2024). Ginsenoside Rg3 further attenuates testicular injury by suppressing OS-driven apoptosis and ferroptosis while preserving Sertoli and Leydig cell function. In dibis (2-ethylhexyl)phthalate/mono- (2-ethylhexyl) phthalate -induced reproductive toxicity models, Rg3 restored antioxidant enzyme activity, reduced lipid peroxidation, improved androgen receptor and follicle-stimulating hormone receptor expression, and reinforced blood–testis barrier integrity via increased zonula occludens-1 (ZO-1) and occludin expression. As these effects were demonstrated under high-dose toxicant exposure rather than the chronic venous and thermal stress characteristic of VC, their relevance to VC remains inferential and requires disease-specific validation (Ma, 2024).

### 3.3 *Rhodiola rosea* L., Crassulaceae (Hongjingtian)

*Rhodiola rosea* L. is a medicinal plant whose dried root and rhizome (Hongjingtian) have been used since it was first recorded in the Compendium of Materia Medica (Bencao Gangmu), completed in 1578. Traditionally, Hongjingtian is used to “benefit qi” (support energy metabolism), promote blood circulation, unblock meridians, and relieve respiratory symptoms such as asthma (Panossian et al., 2010). More than 140 metabolites have been isolated and identified from its roots and rhizomes. These include flavonoids, phenylethanes, phenylpropanoids, phenols, and terpenoids. Among these, salidroside is considered the principal active metabolite and is credited with anti-inflammatory, antioxidant, antitumor, and antiviral activities, as well as protective effects on the cardiovascular, neurological, renal, and hepatic systems (Hung et al., 2011; Bernatoniene et al., 2023).

Hongjingtian has demonstrated significant antioxidant potential through its bioactive metabolites, including glycosides and flavonoids, which exhibit robust free radical scavenging capabilities against •OH, H_2_O_2_, and 1,1-diphenyl-2-picryl-hydrazyl radical (Jówko et al., 2018). In a hypoxia-induced GC-1 germ cell model, the metabolite salidroside (5–10 μM), *Rhodiola rose*a L.-derived metabolites suppressed apoptosis by modulating the Bcl-2/Bax ratio and inhibiting cleaved caspase-3 activation. It effectively preserved mitochondrial integrity by enhancing SOD and ATP levels while reducing ROS and MDA accumulation. These results derive from an *in vitro* hypoxia model and therefore do not by themselves demonstrate efficacy in the complex, chronic hypoxic/hemodynamic environment of VC (Song, 2023). Likewise, Rhodiola polysaccharides have been shown to improve mitochondrial function and acrosomal integrity in cryopreserved sperm via upregulation of SOD/GPX and suppression of lipid peroxidation, but the cryoprotective context and dosing differ substantially from VC pathophysiology, so direct translational claims require validation in appropriate *in vivo* VC models and with standardized extract characterization (Yang et al., 2016).

### 3.4 *Cuscuta chinensis* Lam., Convolvulaceae (Tusizi)

*Cuscuta chinensis* Lam. is a medicinal plant whose dried mature seeds (Tusizi) have been used since it was first recorded in the Shennong’s Classic of Materia Medica (Shennong Bencao Jing) (Donnapee et al., 2014; Wang K. et al., 2022). Flavonoids and polysaccharides have been recognized as predominant metabolites of Tusizi, underpinning its pharmacological actions in reproductive protection, immune regulation, and OS mitigation. Additional metabolites—including phenolic acids, lignans, and volatile oils—constitute secondary bioactive fractions, though their mechanistic contributions remain insufficiently characterized and warrant systematic investigation (Ali et al., 2018).

*Cuscuta chinensis* Lam. exhibits protective effects in chemically induced models of testicular toxicity, but the translational link to VC requires caution. In GC-1 spg cells exposed to Tripterygium glycosides, a *C. chinensis*-containing preparation preserved ultrastructure, inhibited apoptosis, increased S-phase fraction, and enhanced mitochondrial membrane potential; however, this result derives from a multi-botanical drug intervention and a toxicant model rather than isolated Tusizi in VC-specific pathology, and lacks extract standardization and PK information (Wang et al., 2021). In experimental VC rats, *C. chinensis* flavonoids reportedly provided dose-dependent protection comparable to the clinical reference drug aescin, while activating the extracellular regulated protein kinases (ERK)–Nrf2/ARE axis and upregulating NAD(P)H quinone oxidoreductase 1 (NQO1)/HO-1 to attenuate cyclophosphamide-induced Leydig-cell apoptosis (Guo et al., 2022; Liu et al., 2018; Li, 2022). These findings support Nrf2-mediated antioxidant mechanisms, but they are mainly preclinical, often use high or poorly characterized doses, and rarely demonstrate that the active metabolites reach therapeutic concentrations within the testicular microenvironment or cross the blood–testis barrier. Multi-component preparations (e.g., MOTILIPERM) that mitigate ER stress via Nrf2/HO-1 further illustrate mechanistic plausibility (Feng et al., 2025; Soni et al., 2018).

### 3.5 *Gynochthodes officinalis* (F.C.How) Razafim. & B.Bremer, Rubiaceae (Bajitian)

*Gynochthodes officinalis* (F.C.How) Razafim. & B.Bremer is a medicinal plant whose dried root (Bajitian) has been used since it was first recorded in the Shennong’s Classic of Materia Medica (Shennong Bencao Jing) (Donnapee et al., 2014). Traditionally, Bajitian is used to invigorate “kidney yang,” a concept generally associated with enhancing vitality and reproductive function—strengthen tendons and bones, and alleviate symptoms attributed to “wind-dampness,” such as joint stiffness or pain (Zhang et al., 2018). Bajitian contains various metabolites, including anthraquinones, naphthoic acid methyl esters, cyclic enol ether terpenes, pentacyclic triterpenoids, and sugar metabolites, with carbohydrates comprising approximately 50% of its dry weight (Liu et al., 2024b). Bajitian and its active metabolites have pharmacological effects such as antidepressant, antidementia, anti-aging, anti-inflammatory, antioxidant, further supporting its therapeutic potential (Zhang and Zhang, 2022).

The polyherbal preparation DA-9401—composed of *G. officinalis* (F.C.How) Razafim. & B.Bremer (Rubiaceae), *Lilium lancifolium* Thunb. (Liliaceae), and *C. chinensis* Lam. (Convolvulaceae) exerts multi-target protection against VC-induced testicular injury. For example, *G. officinalis* polysaccharides (100–300 mg kg^−1^) produced dose-dependent recovery of seminiferous tubule diameter and sperm counts in rodent VC models while modulating protein tyrosine phosphatase (PTEN)/PI3K/Akt and Nrf2/HO-1 pathways (Yu et al., 2024), but these findings derive from high-dose *in vivo* experiments using incompletely characterized extracts and without PK/PD linkage or routine positive-control comparators, limiting direct translational inference. Mechanistic assays report that DA-9401 components inhibit inositol-requiring enzyme one (p-IRE1α)/c-Jun N-terminal kinase (p-JNK) and upregulate Claudin-11 to mitigate endoplasmic reticulum stress and preserve blood–testis barrier integrity, yet these effects are largely demonstrated in cell or short-term animal models with mixed formulations rather than isolated, standardized fractions (Yuan et al., 2022). *In vitro, G. officinalis* metabolites and related fractions enhanced SOD/catalase (CAT) and supported testosterone secretion in interstitial cells, but the protective concentrations used and the absence of extract standardization and blood–testis barrier penetration data constrain conclusions about *in situ* efficacy (Chang et al., 2008; Zhang, 2017).

### 3.6 *Epimedium brevicornu* Maxim., Berberidaceae (Yinyanghuo)

*Epimedium brevicornu* Maxim is a medicinal plant whose dried leaves (Yinyanghuo) have been used since it was first recorded in the Shennong’s Classic of Materia Medica (Shennong Bencao Jing) (Zheng et al., 2020). Traditionally, Yinyanghuo is used to invigorate kidney yang, strengthen tendons and bones, and alleviate symptoms of “wind-dampness”. A diverse array of chemical metabolites in Yinyanghuo—such as flavonoids, polysaccharides, alkaloids, lignans, and trace elements, which exhibit anti-inflammatory, antioxidant, antitumor, and anti-apoptotic activities, underpinning its clinical application in managing multisystem diseases such as male infertility, osteoporosis, hyperplasia of mammary glands, hematological disorders, and bronchitis (Lei et al., 2025; Ma et al., 2023).

Yinyanghuo formulations demonstrates clinically significant efficacy in addressing VC-associated sperm dysfunction, evidenced by a 61.3% pregnancy rate among partners of patients treated with Yinyanghuo-containing formulations (e.g., Shengjing tablets) over a 3–18-month follow-up period (Chen et al., 2006). However, the absence of a control arm and the presence of substantial confounders—such as concurrent lifestyle interventions and scrotal cooling—preclude definitive attribution of benefit to *E. brevicornu per se*. Preclinical data implicate several *E. brevicornu*-derived metabolites in redox modulation: icaritin and icariin enhance SOD and GSH peroxidase activities, reducing MDA levels, and scavenge ROS, thereby improving the epididymal microenvironment. Icaritin modulates the Nrf2/Keap1/HO-1 signaling pathway to enhance cellular antioxidant defenses, while icariin inhibits hypoxia-induced apoptosis in epididymal epithelial cells of patients with VC (Zha et al., 2014a; Huang et al., 2022). Additional studies suggest icariin downregulates pro-apoptotic p53, relieves suppression of solute carrier family 7 member 11 (SLC7A11), promotes glutathione synthesis and GPX4 activity, thereby inhibiting ferroptosis and preserving testicular cell integrity; however, these data derive from chemically induced reproductive-toxicity models rather than VC models and employed relatively high systemic doses without pharmacokinetic justification (Zha et al., 2014b; Jiang et al., 2024). Moreover, Yinyanghuo upregulates NO production via the phospholipase C gamma 1 (PLCγ1)/Akt/eNOS signaling pathways, improving testicular microcirculation and addressing oligozoospermia in rats, but these experiments likewise used high systemic dosing and lacked tissue pharmacokinetic data to support translational relevance (Deng et al., 2023).

### 3.7 *Schisandra chinensis* (Turcz.) Baill., Schisandraceae (Wuweizi)

*Schisandra chinensis* (Turcz.) Baill is a medicinal plant whose dried mature fruit (Wuweizi) has been used since it was first recorded in the Shennong’s Classic of Materia Medica (Shennong Bencao Jing) (Yang et al., 2022; Lu and Chen, 2009). Phytochemically, Wuweizi contains lignans, volatile oils, terpenes, organic acids, flavonoids, polysaccharides, and inorganic elements. Among these, lignans are regarded as the principal active metabolites, with representative metabolites such as schisandra A, schisandra B, and schisandra C (Olas, 2023). These profiles underpin its reported therapeutic benefit across liver disorders, neurodegenerative disease, metabolic disturbance, and male infertility management (Zhou et al., 2021).

Wuweizi exerts redox-modulating properties largely via lignans and flavonoids that possess radical-scavenging activity (Kopustinskiene and Bernatoniene, 2021). In VC, schisandra B has been shown to activate the sirtuin 1 (SIRT1)/Nrf2 signaling pathway and to upregulate mitochondrial antioxidant defenses by increasing SOD2 upregulation and GSH expression, thereby reducing ROS accumulation. Functionally, these actions have translated into improved human sperm motility *in vitro* and rescue of mouse blastulation rates under experimentally induced OS. However, these findings fail to account for the chronic hemodynamic pathology of VC or verify the metabolite’s pharmacokinetic ability to cross the blood-testis barrier *in vivo* (Zhang et al., 2023).

### 3.8 *Lycium chinense* Mill., Solanaceae (Gouqi)

*Lycium chinense* Mill. is a medicinal plant whose dried mature fruit (Gouqi) has been used since it was first recorded in the Shennong’s Classic of Materia Medica (Shennong Bencao Jing) (Potterat, 2010).Traditionally, Gouqi is used to nourish kidney yin, moisten the lungs for arresting cough, and nourish the liver for improving eyesight (Ma et al., 2023). Gouqi contains over 200 identified chemical metabolites, with polysaccharides, flavonoids, fat-soluble pigments, and vitamins being the primary classes. Among these, *L. chinense* polysaccharides, betaine, and wolfberry pigments are recognized as the main active metabolites. Extensive studies have demonstrated that Gouqi exerts diverse pharmacological effects, including enhanced immune regulation, antioxidant, anti-aging, antitumor, and the regulation of blood sugar and lipid levels (Yao et al., 2021; Jeong et al., 2020).

*Lycium chinense* polysaccharide (LCP), a water-soluble polysaccharide that exerts antioxidant effects by direct radical scavenging, potentiation of endogenous antioxidant enzymes and activation of cytoprotective signaling. In VC, LCP reduced lipid peroxidation (↓MDA), increased SOD/CAT/GSH, attenuated caspase-dependent apoptosis, and improved cellular viability—effects that translated into improved sperm quality indices in some studies (Yang et al., 2024; Xu, 2021; Tian, 2022). Notably, LCP upregulated GPX4 and suppressed AIF in aging-related oxidative injury models, suggesting protection against ferroptosis and mitochondrial dysfunction (Liu C. L. et al., 2021). However, many positive findings derive from acute chemical or cell-culture oxidative-stress paradigms (e.g., hypoxanthine–xanthine oxidase systems, GC-1 spg cells) that recapitulate superoxide burst but do not model the chronic hyperthermia, venous stasis and metabolic hypoxia characteristic of VC. Dosing regimens and systemic exposure used in animal experiments are often high and lack pharmacokinetic justification. Thus, while mechanistically plausible, the specific relevance of existing LCP data to VC pathophysiology remains provisional.

### 3.9 *Curcuma longa* L., Zingiberaceae (Jianghuang)

*Curcuma longa* L. is a medicinal plant whose dried rhizome (Jianghuang) has been used since it was first recorded in the Newly Revised Materia Medica (Xinxiu Bencao), written in the Tang dynasty (618–907 CE) (Zhu et al., 2023). Traditionally, Jianghuang is used to relieve stasis and activate qi, and promote menstruation to relieve pain (Govindarajan, 1980). The primary chemical metabolites of Jianghuang include curcuminoids, volatile oils, flavonoids, sugars, alkaloids, organic acids, and inorganic elements (Ammon and Wahl, 1991). Curcuminoids and volatile oil metabolites are regarded as the main pharmacological agents. Their diverse activities, including anti-inflammatory, antitumor, antioxidant, antibacterial, and hypoglycemic effects, provide a robust theoretical foundation for the use of turmeric in the management of neurological, gastrointestinal, and cardiovascular disorders (Aggarwal and Harikumar, 2009; Alizadeh et al., 2018).

Under VC-associated testicular heat stress, curcumin attenuates spermatocyte apoptosis by suppressing activating transcription factor 3 (Atf3). This inhibition reduces Atf3 binding at the Bcl-2 promoter, thereby upregulating Bcl-2 expression and, in parallel, enhancing cellular antioxidant and anti-inflammatory defenses. Notably, this 38 °C thermal model effectively replicates the scrotal hyperthermia component of VC, but the clinical translation of curcumin is chronically hampered by its poor aqueous solubility and low systemic bioavailability (Liu, 2024). Moreover, ionizing radiation (an acute, high-energy exogenous insult) induces ROS overproduction and intracellular redox imbalance, and both *in vitro* and *in vivo* studies show that curcumin can counteract radiation-triggered OS via activation of the Nrf2/HO-1 pathway. Upregulation of Nrf2 thereby enhances cellular resilience to oxidative injury and likely contributes to curcumin’s cytoprotective profile. Unlike the acute, high-energy exogenous insult of ionizing radiation, VC-associated injury is a chronic, endogenous process driven by venous stasis and thermal stress (Yang, 2018). Additionally, at 50 mg/kg, curcumin ameliorates cyclophosphamide-induced reproductive toxicity in mice by restoring testicular antioxidant capacity (increasing GSH and SOD) and reducing lipid peroxidation markers, further supporting its testis-protective potential (Yang, 2016).

### 3.10 *Rheum palmatum* L., Polygonaceae (Dahuang)

*Rheum palmatum* L. is a medicinal plant whose dried root and rhizome (Dahuang) have been used since it was first recorded in the Shennong’s Classic of Materia Medica (Shennong Bencao Jing) (Xu et al., 2024). Traditionally, Dahuang is used to remove accumulation with purgation, clear heat fire, cool the blood, and detoxify, and eliminate blood stasis to promote menstruation (Zheng et al., 2021). Because raw Dahuang can impair spleen and stomach function, it is commonly processed (e.g., wine-processed, cooked, or charred forms), which modifies its chemical profile, attenuates gastrointestinal irritation, and enhances therapeutic applicability (Qiu et al., 2024). Recent investigations have focused on the active components of Dahuang, particularly anthraquinones, dianthrones, tannins, and polysaccharides, which exhibit pharmacological activities such as antibacterial, anti-inflammatory, antitumor, and lipid-lowering effects (Wang et al., 2023). In a rat model of VC, emodin intervention (20 mg/kg/d, intraperitoneal) suppresses apoptosis of spermatogenic epithelial cells, reduces the expression of pro-apoptotic proteins, lowers OS levels, and mitigates structural damage to the seminiferous tubules, thereby protecting normal spermatogenesis in the testis (Nie, 2021). Notably, this study employed dual-model validation (utilizing both the surgical left renal vein ligation and a GC-1 cell hypoxia model), which directly addresses the hypoxic component of VC pathology. Nevertheless, the exclusive use of intraperitoneal delivery constrains clinical extrapolation, as it circumvents first-pass metabolism and gastrointestinal absorption inherent to oral administration.

## 4 The TCM diagnostic and therapeutic rationale for varicocele

TCM employs a pattern-based diagnostic framework in which treatment is guided by the identification of symptom complexes (“zheng” in Chinese), rather than by a single disease entity. In this approach, individualized botanical prescriptions are formulated by combining multiple botanical drugs with complementary functions, and dosages are adjusted according to the patient’s constitution and clinical presentation (Xu et al., 2019). Within this clinical framework, VC is not viewed solely as a vascular disorder but is understood as a complex syndrome involving multiple TCM organs and functional systems. Central to this view is the concept of “Jing Shi”, a complex concept in TCM physiology that may be translated as the male reproductive system, including the testes, epididymis, prostate, and related structures. This system governs semen storage, fertility, and reproduction, and is closely associated with kidney and liver functions in TCM (Guo, 2021; Ma et al., 2024; Zhong et al., 2015). According to TCM pathophysiology, VC is commonly categorized into patterns reflecting a mixture of excess and deficiency features. Early stages are often characterized by qi stagnation and blood stasis; intermediate stages may involve blood stasis and damp-heat; and latter stagesfrequently present with underlying deficiencies of kidney yang, liver-kidney yin, or qi. Consequently, therapeutic principles emphasize reinforcing circulation and resolving stasis in earlier phases, while incorporating tonifying approaches in chronic or advanced presentations, thereby aligning treatment selection with the patient’s predominant clinical pattern (See Table 3; Figure 3).

**TABLE 3:** Category of effects, composition, pathogenesis and origin of multi-botanical drug formulations.

Category of effects	Multi-botanical drug formulations	Composition	Pathogenesis	Origin	Ref.
Activating blood circulation for removing blood stasis	GuiZhi FuLing Wan	*Cinnamomum cassia* (L.) J.Presl [Lauraceae] (Guizhi), *Wolfiporia extensa* (Peck) Ginns [Polyporaceae] (Fuling), *Prunus persica* (L.) Batsch [Rosaceae] (Taoren), *Paeonia lactiflora* Pall. [Paeoniaceae] (Baishao), and *Paeonia suffruticosa* Andrews [Paeoniaceae] (Mudanpi)	Qi stagnation and blood stasis in the lower jiao	Synopsis of the Golden Cabinet (Jingui Yaolüe)	Tanaka et al., 2021 Zhang (2022)
Invigorating spleen-stomach and replenishing qi	Buzhong Yiqi decoction	*Astragalus mongholicus* Bunge [Fabaceae] (Huangqi), *Atractylodes macrocephala* Koidz. [Asteraceae] (Baizhu), *Glycyrrhiza uralensis* Fisch. ex DC. [Fabaceae] (Gancao), *Panax ginseng* C.A.Mey. [Araliaceae] (Renshen), *Citrus reticulata* Blanco [Rutaceae] (Chenpi), *Bupleurum chinense* DC. [Apiaceae] (Chaihu), and *Cimicifuga foetida* L. [Ranunculaceae] (Shengma)	Qi deficiency depression syndrome	Treatise on spleen and stomach (Piwei lun)	Wang et al., 2022 Li (2019)
Activating blood circulation for removing obstruction in collaterals	Dahuang Zhechong Wan	*Rheum palmatum* L. [Polygonaceae] (Dahuang), *Eupolyphaga sinensis* Walker [Corydiidae] (Zhechong), *Prunus persica* (L.) Batsch [Rosaceae] (Taoren), *Scutellaria baicalensis* Georgi [Lamiaceae] (Huangqin), *Prunus armeniaca* L. [Rosaceae] (Xingren), *Hirudo nipponia* Whitman [Hirudinidae] (shuizhi), *Tabanus bivittatus* Matsumura [Tabanidae] (Mengchong), *Paeonia lactiflora* Pall. [Paeoniaceae] (Baishao), *Holotrichia diomphalia* Bates [Scarabaeidae] (Qicao), *Rehmannia glutinosa* (Gaertn.) DC. [Orobanchaceae] (Dihuang), *Toxicodendron vernicifluum* (Stokes) F.A.Barkley [Anacardiaceae] (Ganqi) and *Glycyrrhiza uralensis* Fisch. ex DC. [Fabaceae] (Gancao)	Static blood obstruction and stagnation	Essentials of the Golden Cabinet (Jingui Yaolüe)	An et al. (2013)
Dispersing stagnated liver qi for removing obstruction in collaterals	Tong Jing Ling	*Bupleurum chinense* DC. [Apiaceae] (Chaihu), *Carthamus tinctorius* L. [Asteraceae] (Honghua), *Salvia miltiorrhiza* Bunge [Lamiaceae] (Danshen), *Panax notoginseng* (Burkill) F.H.Chen [Araliaceae] (Sanqi), *Cuscuta chinensis* Lam. [Convolvulaceae] (Tusizi), *Lycium barbarum* L. [Solanaceae] (Gouqi), and *Eleutherococcus nodiflorus* (Dunn) S.Y.Hu [Araliaceae] (Wujiapi), together with animal- and mineral-derived materials such as “dragon bone” (Longgu; fossil bone, mineral drug), *Ostrea* spp. (Ostreidae; Muli), and *Polistes mandarinus* de Saussure (Vespidae; Lufengfang)	Liver depression and kidney deficiency	Hospital formulations (Ningbo Municipal Hospital of TCM)	Cui et al. (1998)
Clearing heat and removing toxicity	Mailuo Shutong pills	*Astragalus mongholicus* Bunge [Fabaceae] (Huangqi), *Lonicera japonica* Thunb. [Caprifoliaceae] (Jinyinhua), *Phellodendron chinense* C.K.Schneid. [Rutaceae] (Huangbo), *Atractylodes lancea* (Thunb.) DC. [Asteraceae] (Cangzhu), *Coix lacryma-jobi* L. [Poaceae] (Yiyiren), *Scrophularia ningpoensis* Hemsl. [Scrophulariaceae] (Xuanshen), *Angelica sinensis* (Oliv.) Diels [Apiaceae] (Danggui), *Paeonia lactiflora* Pall. [Paeoniaceae] (Baishao), *Glycyrrhiza uralensis* Fisch. ex DC. [Fabaceae] (Gancao)	Blockade of damp-heat and static blood	Approved by CFDA (Z20090636)	Wang et al. (2023)

**FIGURE 3:**
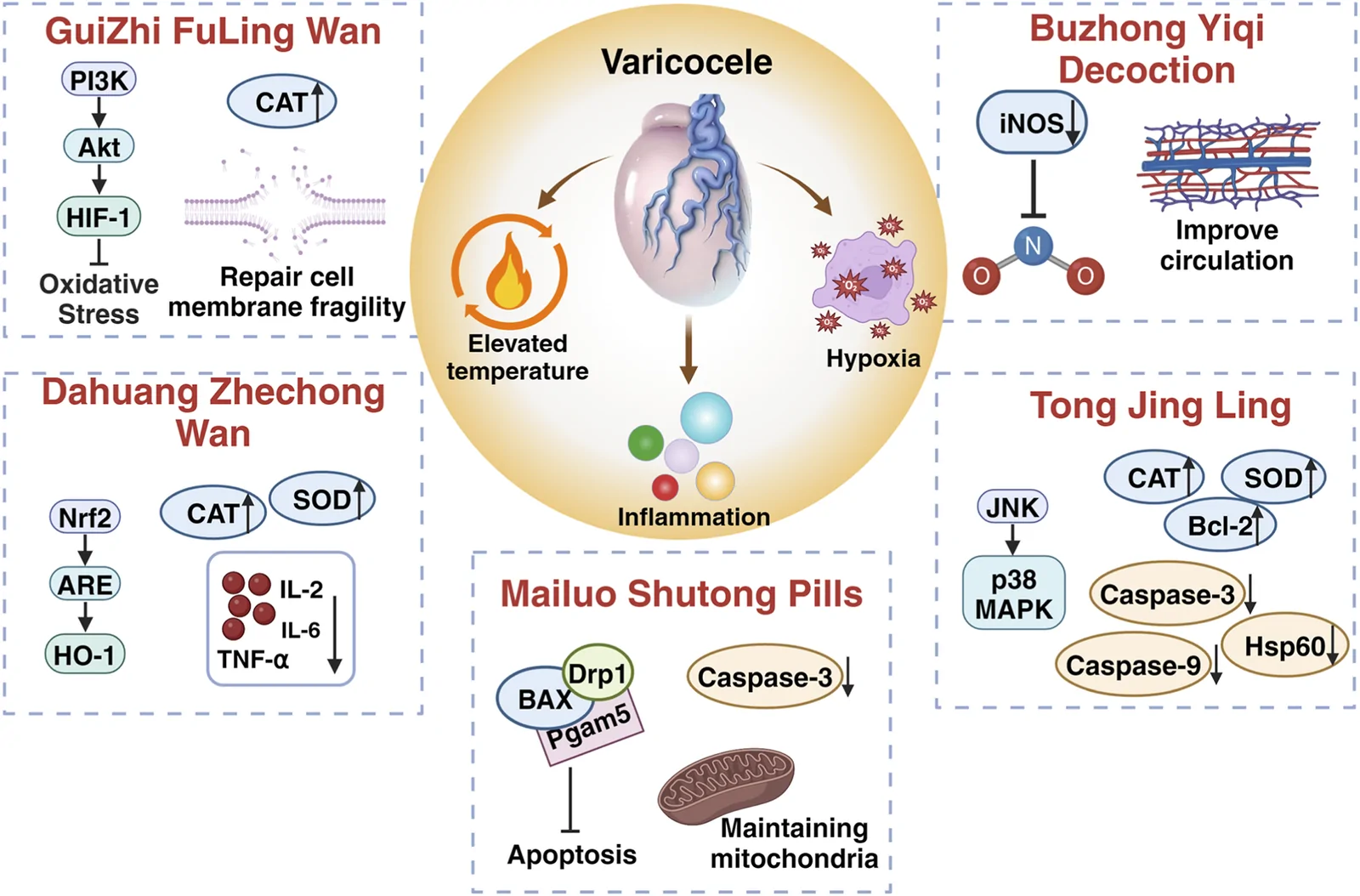
Botanical drugs and major plant-derived metabolites with potential relevance to varicocele and putative mechanisms of action. Varicocele-associated venous stasis produces scrotal hyperthermia, local hypoxia and inflammatory activation that together increase reactive oxygen species, impair mitochondrial function, and trigger apoptosis and adverse semen parameters; the boxed interventions are shown with their reported molecular targets and functional effects: GuiZhi FuLing Wan attenuates oxidative stress via activation of the PI3K/Akt/HIF-1 axis, increases catalase activity and helps repair cell-membrane fragility; Buzhong Yiqi Decoction improves microcirculation and reduces iNOS–mediated nitric oxide production; Dahuang Zhechong Wan activates the Nrf2/ARE/HO-1 antioxidant pathway, upregulates SOD/CAT and lowers pro-inflammatory cytokines (IL-2, IL-6, TNF-α); Mailuo Shutong Pills inhibit mitochondria-mediated apoptotic signaling (BAX/Drp1/PGAM5), decrease caspase-3 activation and help preserve mitochondrial integrity; and Tong Jing Ling suppresses stress-kinase signalling (JNK → p38 MAPK), increases antioxidant proteins (SOD, CAT) and Bcl-2 while downregulating caspase-3/9 and Hsp60 to limit apoptosis. Collectively, these multi-target actions converge on reducing oxidative injury, inflammation and cell death, thereby supporting mitochondrial function and improving testicular/sperm outcomes in varicocele.

### 4.1 Multi-botanical drug formulations

#### 4.1.1 GuiZhi FuLing Wan (GFW)

Guizhi Fuling Wan (GFW) is a multi-botanical drug preparation composed of *Cinnamomum cassia* (L.) J.Presl [Lauraceae] (Guizhi), *Wolfiporia extensa* (Peck) Ginns [Polyporaceae] (Fuling), *Prunus persica* (L.) Batsch [Rosaceae] (Taoren), *Paeonia lactiflora* Pall. [Paeoniaceae] (Baishao), and *Paeonia suffruticosa* Andrews [Paeoniaceae] (Mudanpi), originally documented in Jingui Yaolüe (Synopsis of the Golden Cabinet) by Zhang Zhongjing during the Eastern Han dynasty (Tanaka et al., 2021; Wang Q. et al., 2022). In contemporary clinical practice, GFW has been included in the China Sexology Association guideline as a recommended botanical intervention for VC-associated conditions (Wang and Dai, 2025).

The clinical efficacy of GFW in VC management has been reported (Ishikawa et al., 1996), with its derivative formulation showing grade-dependent improvements in semen quality (74% in grade I, 58.05% in grade II, and 28.22% in grade III). However, this study involved a small, non-randomized clinical cohort without standardized outcome measures or placebo/active comparators, and the botanical preparation was not chemically characterized. Synergistic enhancement of therapeutic outcomes has been observed when GFW is adjunctively administered with laparoscopic varicocelectomy, with greater improvements in sperm density, motility, and morphology compared with surgery alone after 6 months of follow-up (Wei et al., 2010). Yet, this evidence derives from a single, non-blinded study using a combined botanical drug regimen, which limits attribution of the effects specifically to GFW. Mechanistic investigations further reveal GFW may mitigate ROS-mediated cellular injury by reducing erythrocyte membrane fragility and preserving vascular integrity, thereby improving hemodynamics associated with VC-related microcirculatory disturbance (Wang et al., 2015). Additional studies reported endothelial regulatory effects, including enhanced acetylcholine-mediated vasodilatation, suppression of xanthine oxidase and lipoxygenases activity, and modulation of eNOS/iNOS and vascular cell adhesion protein one in preclinical models (Sekiya et al., 2002). However, these data were obtained in non-VC vascular injury models using whole-formula extracts, without disease-specific models or pharmacokinetic characterization. Network pharmacology and molecular docking analyses further propose that GFW targets OS-related pathways, particularly PI3K-Akt and HIF-1 signaling, which regulate redox homeostasis, apoptosis, and angiogenesis, but these *in silico* findings require experimental validation. Identified metabolites such as quercetin, kaempferol, and baicalein of GFW demonstrate potent free radical scavenging capacity, although their concentrations, bioavailability, and activity within the VC-affected testicular microenvironment remain insufficiently characterized (Nozaki et al., 2007).

#### 4.1.2 Buzhong Yiqi Decoction (BYD)

Buzhong Yiqi Decoction (BYD) is a classical multi-botanical preparation composed of *A. mongholicus* Bunge [Fabaceae] (Huangqi), *Atractylodes macrocephala* Koidz. [Asteraceae] (Baizhu), *Glycyrrhiza uralensis* Fisch. ex DC. [Fabaceae] (Gancao), *P. ginseng* C.A.Mey. [Araliaceae] (Renshen), *Citrus reticulata* Blanco [Rutaceae] (Chenpi), *Bupleurum chinense* DC. [Apiaceae] (Chaihu), and *Cimicifuga foetida* L. [Ranunculaceae] (Shengma). The formula was originally described in Piwei Lun (Treatise on the Spleen and Stomach) by Li Dongyuan in 1249. Traditionally, it is used to address conditions interpreted in TCM as “qi deficiency”, a concept often associated with reduced physiological resilience and impaired microcirculation. In contemporary practice, BYD has been applied in the management of conditions such as VC, where impaired circulation and testicular dysfunction are prominent features (Li et al., 2022).

Clinical investigations have validated the potential therapeutic benefits of BYD in primary VC, with a longitudinal study (n = 38) reporting symptomatic improvement in testicular pain in 89.47% of patients, including 55.26% with marked improvement and 34.21% with moderate responses (Dong et al., 2024). This study was based on a small, single-arm cohort without randomization, placebo or active comparators, and relied primarily on subjective symptom scores, which limits generalizability of the findings. Similarly, a 3-month observational study (n = 38) using a modified BYD reported an overall response rate of 89.47% (Guan et al., 2011), but the absence of randomization, comparator groups, and standardized reproductive endpoints constrains interpretation of its effects on semen quality or postoperative outcomes. At the mechanistic level, VC is associated with iNOS activation and excessive NO production, which may impair spermatogenesis, whereas physiological eNOS-derived NO exerts vasodilatory and effects. Experimental studies suggest that BYD may modulate this balance, reducing NO levels and improving testicular function in rat models, potentially through enhanced eNOS activity and reduced inflammatory cell adhesion (Wang, 2017; Förstermann et al., 2017).

#### 4.1.3 Dahuang Zhechong Wan (DZW)

Dahuang Zhechong Wan (DZW) is a classical multi-component botanical–animal preparation composed of *R. palmatum* L. [Polygonaceae] (Dahuang), *Eupolyphaga sinensis* Walker [Corydiidae] (Zhechong), *P. persica* (L.) Batsch [Rosaceae] (Taoren), *Scutellaria baicalensis* Georgi [Lamiaceae] (Huangqin), *Prunus armeniaca* L. [Rosaceae] (Xingren), *Hirudo nipponia* Whitman [Hirudinidae] (Shuizhi), *Tabanus bivittatus* Matsumura [Tabanidae] (Mengchong), *P. lactiflora* Pall. [Paeoniaceae] (Baishao), *Holotrichia diomphalia* Bates [Scarabaeidae] (Qicao), *Rehmannia glutinosa* (Gaertn.) DC. [Orobanchaceae] (Dihuang), *Toxicodendron vernicifluum* (Stokes) F.A.Barkley [Anacardiaceae] (Ganqi) and *G. uralensis* Fisch. ex DC. [Fabaceae] (Gancao). The formula was first recorded in Jingui Yaolüe (Synopsis of the Golden Cabinet) by Zhang Zhongjing during the Eastern Han dynasty (25-220CE) (An et al., 2013). Traditionally, it is used for conditions interpreted as impaired microcirculation or tissue perfusion. In contemporary practice, DZW has been recommended by the China Sexology Association for the management of VC, particularly in patients presenting with symptoms consistent with circulatory disturbance (Cai et al., 2010).

DZW has been evaluated in adolescent VC complicated by oligozoospermia and testicular volume abnormalities. In a randomized active-controlled study, DZW (3 g twice daily) improved sperm concentration, progressive/total motility, and endocrine indices (↑total testosterone, ↓prolactin) with a reduced testicular atrophy index (within-group P < 0.05), but it was not superior to the comparator (aescin) between groups, and the short duration, adolescent-only population, and lack of OS biomarkers constrain mechanistic inference and generalization to adult VC (Mo, 2021). Preclinical studies using modified DZW granules in VC rats report improved epididymal sperm quality and partial restoration of testicular/epididymal histopathology and ultrastructure, alongside higher antioxidant-related readouts and increased Nrf2/HO-1 expression and reduced inflammatory cytokine transcripts (IL-2, IL-6, TNF-α), with aescin included as an active comparator in some designs (Wang et al., 2017; Meng, 2021; Jiang et al., 2021). Collectively, these data are consistent with engagement of an Nrf2–HO-1–linked cytoprotective program that may counteract oxidative injury and apoptosis; however, pathway activation is largely inferred from expression endpoints without upstream Keap1–Nrf2 dynamics, nuclear translocation, downstream ARE gene panels, or functional perturbation, and dosing/formulation differences (including very high gavage doses) limit comparability with clinically used preparations.

#### 4.1.4 Tong Jing Ling (TJL)

Tianjing Liujiu (TJL) is a modern TCM multi-component preparation developed by Ningbo Municipal Hospital of Traditional Chinese Medicine and includes *B. chinense* DC. [Apiaceae] (Chaihu), *Carthamus tinctorius* L. [Asteraceae] (Honghua), *Salvia miltiorrhiza* Bunge [Lamiaceae] (Danshen), *Panax notoginseng* (Burkill) F.H.Chen [Araliaceae] (Sanqi), *C. chinensis* Lam. [Convolvulaceae] (Tusizi), *Lycium barbarum* L. [Solanaceae] (Gouqi), and *Eleutherococcus nodiflorus* (Dunn) S.Y.Hu [Araliaceae] (Wujiapi), together with animal- and mineral-derived materials such as “dragon bone” (Longgu; fossil bone, mineral drug), *Ostrea* spp. (Ostreidae; Muli), and *Polistes mandarinus* de Saussure (Vespidae; Lufengfang) (Cui et al., 1998). In clinical practice, TJL is used for symptom patterns interpreted as microcirculatory disturbance and chronic discomfort, and has been applied in VC management in local clinical settings.

In VC, OS is closely linked to sperm DNA damage and germ-cell loss; accordingly, TJL has been evaluated primarily for its ability to restore redox balance and preserve sperm genomic integrity. *In vivo*, a surgically induced Wistar rat VC model showed that 8-week TJL treatment reduced testicular H_2_O_2_ and lowered epididymal DNA fragmentation index (DFI) *versus* the untreated model group, supporting an association between improved redox homeostasis and DNA integrity (Du et al., 2017); however, the added “tail-clamping irritation” stress paradigm, absence of a pharmacological antioxidant positive control, and lack of extract standardization limit attribution of the DFI benefit to a specific testis-targeted antioxidant action. Dose–response evidence further suggests a threshold pattern: in a VC rat study using 0.6/1.2/2.4 g mL^-1^ decoction for 8 weeks, mitochondrial endpoints (ROS↓, matrix metalloproteinases↑) improved mainly at medium/high doses (approaching an L-carnitine control), whereas the low dose showed limited separation (Liu et al., 2018; Cun et al., 2009). Clinically, a 3-month randomized outpatient study (n = 65) reported improvements in motility/morphology and DFI in both arms, with TJL achieving only a modestly lower post-treatment DFI than aescin + Wuzhiyanzong pills and no clear superiority in bulk semen parameters, suggesting a limited incremental effect beyond comparator/background improvement (Wu et al., 2016). Mechanistically, TJL reduced germ-cell apoptosis and increased Bcl-2 in VC rats, and in another VC model combined with stress, it attenuated p-JNK/p-p38 with concomitant reductions in Bax/Cyt-c/AIF, compatible with suppression of a ROS-driven JNK/p38→mitochondrial apoptosis; nevertheless, the pathway inference remains largely correlative because key nodes were not reported (Cun et al., 2008; Liu et al., 2019; Zheng, 2017).

### 4.2 Patent pills

#### 4.2.1 Mailuo Shutong Pills (MLST)

Mai Luo Shu Tong (MLST; CFDA approval No. Z20090636) is a multi metabolite botanical-based patent medicine used clinically in China for conditions characterized by microcirculatory disturbance and inflammatory burden. Its plant-derived metabolites include *A. mongholicus* Bunge [Fabaceae] (Huangqi), *Lonicera japonica* Thunb. [Caprifoliaceae] (Jinyinhua), *Phellodendron chinense* C.K.Schneid. [Rutaceae] (Huangbo), *Atractylodes lancea* (Thunb.) DC. [Asteraceae] (Cangzhu), *Coix lacryma-jobi* L. [Poaceae] (Yiyiren), *Scrophularia ningpoensis* Hemsl. [Scrophulariaceae] (Xuanshen), *Angelica sinensis* (Oliv.) Diels [Apiaceae] (Danggui), *P. lactiflora* Pall. [Paeoniaceae] (Baishao), *G. uralensis* Fisch. ex DC. [Fabaceae] (Gancao). In addition, according to the package insert, the animal-derived metabolites are listed by medicinal common names, including wugong (centipede), shuizhi (medicinal leech), and quanxie (scorpion); species-level identification should follow the corresponding pharmacopoeial/label specification for the product batch. MLST is listed in the 2020 edition of the Pharmacopoeia of the People’s Republic of China (Wang et al., 2023).

MLST pills been reported to improve postoperative recovery in VC. In a single-center randomized study (n = 120; all patients underwent laparoscopic high ligation), adjunct MLST (vs. aspirin) was associated with higher reported “cure” rates and better semen indices at 6 months, alongside fewer postoperative complications (Liu and Hou, 2020). Because the control was aspirin rather than placebo/standard postoperative care and key outcomes (e.g., “cure”/recurrence) were not clearly standardized, the specificity and magnitude of benefit should be interpreted cautiously. Mitochondria, which serve as a primary site for ROS generation in sperm, especially through the electron transport chain, play a critical role in this context. VC-associated redox imbalance exacerbates OS, excessive ROS not only induces aberrant modifications in mitochondrial DNA and structure but also precipitates mitochondrial dysfunction, thereby triggering intrinsic (mitochondrial) apoptotic pathways. Consistent with this framework, a VC rat study showed that 4-week MLST treatment (initiated 7 days post-modeling) partially restored testicular/epididymal histoarchitecture and mitochondrial ultrastructure, and reduced dynamin-related protein 1 (Drp1)/Bax as well as caspase-9/caspase-3 signals relative to untreated VC rats—consistent with suppression of mitochondria-linked intrinsic apoptosis. However, mechanistic attribution remains inferential because mitochondrial function/ROS and apoptosis were not directly quantified, and as a multi-component patent formula the active metabolites and testicular exposure are unresolved (Jia et al., 2024).

## 5 Conclusions and perspectives

VC is a prevalent male urogenital disorder with a multifactorial pathogenesis involving OS, inflammation, hypoxia, and endocrine dysregulation, and its association with male infertilityis well established. OS is a key pathogenic axis: excessive ROS promote DNA damage, lipid peroxidation, and protein oxidation, thereby disrupting the spermatogenic microenvironment and contributing to reduced sperm motility, increased DFI, abnormal morphology, and, in some patients, scrotal pain/discomfort. Surgical repair remains the primary treatment for VC, particularly for infertility-related VC. Nevertheless, surgery may be inappropriate for subclinical VC or for individuals without reproductive goals. Pharmacological strategies—including venoactive agents like aescuven forte and diosmetin, as well as antioxidant supplementation—have shown variable efficacy and sometimes modest benefits, highlighting the need for adjunctive or alternative options.

Emerging evidence suggests that botanical drugs and plant-derived metabolites may provide complementary, multi-target pharmacological effects relevant to VC. Representative prescriptions such as GFW, BYD, DZW, and TJL have been reported to protect spermatogenesis in preclinical models and to modulate OS-related pathways (e.g., PI3K/Akt, HIF-1), while improving microcirculation indices and attenuating apoptosis. At the metabolite level, candidates such as astragaloside IV, ginsenoside Rg3, salidroside, and curcumin exhibit antioxidant and cytoprotective activities in relevant cellular or animal models. Importantly, these effects cannot be generalized to botanical drugs as a whole and should be interpreted in relation to clearly defined botanical drugs/preparations, standardized extracts, dose–exposure relationships, and model relevance.

Despite mechanistic plausibility, the evidence base remains constrained. Current research disproportionately focuses on infertility-related outcomes, whereas non-reproductive manifestations (e.g., pain and quality-of-life endpoints) and long-term safety receive comparatively limited attention. Many studies are preclinical, use heterogeneous disease models and comparators, and lack extract standardization, pharmacokinetic data, and rigorous endpoints. Clinical evidence is often derived from small, single-center cohorts with limited blinding and non-placebo controls, and a portion of the literature is not readily accessible to an international readership, complicating independent appraisal and replication.

Future work should move from plausibility-driven narratives to hypothesis-tested evidence by matching study design to the clinical heterogeneity of VC and to pharmacological standards. Well-powered clinical trials are needed with standardized eligibility criteria and clinically meaningful endpoints, including validated pain outcomes, semen parameters and reproductive endpoints when relevant, recurrence, and safety, supported by objective biomarkers of OS and inflammation to strengthen biological attribution. In parallel, the botanical material must be defined rigorously through taxonomic authentication, transparent sourcing, and reproducible chemical characterization with marker metabolites to ensure batch consistency. Translational studies should then link exposure to response by clarifying whether key metabolites reach effective concentrations in the testicular and epididymal microenvironment. Mechanistic claims should be validated in VC-relevant models using causal pathway interrogation rather than associative marker changes.
